# Word Mode: a crowding-free reading protocol for individuals with macular disease

**DOI:** 10.1038/s41598-018-19859-0

**Published:** 2018-01-19

**Authors:** Stuart Wallis, Yit Yang, Stephen J. Anderson

**Affiliations:** 1 School of Life & Health Sciences, Aston University, Birmingham, B4 7ET UK; 2 Royal Wolverhampton NHS Trust, New Cross Hospital, Wolverhampton, WV10 0QP UK

## Abstract

Central retinal loss through macular disease markedly reduces the ability to read largely because identification of a word using peripheral vision is negatively influenced by nearby text, a phenomenon termed visual crowding. Here, we present a novel peripheral reading protocol, termed *Word Mode*, that eliminates crowding by presenting each word in isolation but in a position that mimics its natural position in the line of text being read, with each new word elicited using a self-paced button press. We used a gaze-contingent paradigm to simulate a central scotoma in four normally-sighted observers, and measured oral reading speed for text positioned 7.5° in the inferior field. Compared with reading whole sentences, our crowding-free protocol increased peripheral reading speeds by up to a factor of seven, resulted in significantly fewer reading errors and fixations per sentence, and reduced both the critical print size and the text size required for spot reading by 0.2–0.3 logMAR. We conclude that the level of reading efficiency afforded by the crowding-free reading protocol Word Mode may return reading as a viable activity to many individuals with macular disease.

## Introduction

Retaining the ability to read is often reported as the primary concern of individuals seeking visual rehabilitation^1,2^ primarily because impaired reading reduces both quality of life^3^ and the perceived ability to perform daily living tasks^4^. Individuals with macular dysfunction tend to suffer the greatest difficulty with reading because they must rely on their peripheral vision. Depending on the extent of central visual loss, reading with peripheral vision varies from difficult to impossible. There are various reasons why this is so and they are briefly discussed below, together with some of the more popular perceptual manipulations employed to counter the problems associated with reading eccentrically-viewed text.

First, the rapid decline in visual acuity with increasing retinal eccentricity^5,6^ can only be partially compensated by increasing text size^7–12^. This is so because: (i) increased magnification is accompanied by a reduced field of view, which impinges on the minimum ‘textual window’ required for optimal reading^13^ and magnification impairs page navigation ability^14^. Moreover, the dynamic magnification of parafoveal text has been shown to provide no increase in reading speed compared with normal text^15^.

The belief that poor oculomotor control also may contribute to slow peripheral reading speeds^16–18^ (though inconclusive^11,19,20^) led to the development of reading protocols that aimed to minimize the need for saccadic eye movements, such as Rapid Serial Visual Presentation (RSVP). With a standard RSVP protocol, each word is presented sequentially at a regular temporal interval and at a fixed spatial location in the visual field^17,21,22^ (see also^23^). However, although eye movements are dramatically curtailed with this technique, reading speeds are necessarily limited by the temporal crowding effects of forward and backward masking^24,25^, and by individual differences in the temporal processing times of eccentrically-viewed-text^26^. Modifications to the standard protocol that have proved beneficial include: (i) varying word duration with word length^27^; and (ii) allowing reading to proceed at a self-paced manner^28^.

The region around the point of fixation within which a letter can be resolved, termed the visual span^29^, has long been recognized as an important factor in determining reading speed. Legge *et al*.^30^ reported visual spans of less than two letters in peripheral vision, compared with ten letters in central vision, and concluded that reduced visual span was a major factor limiting peripheral reading speeds. Legge *et al*.^31^ went on to argue that visual span length may be jointly determined by the reduced letter acuity and increased crowding evident in the peripheral retina.

Indeed, visual crowding may be one of the most significant factors that limit the ability to read eccentrically-viewed text^12,32–38^. Chung^34,39^ examined the effects of crowding by increasing letter spacing and vertical word spacing. Exaggerated letter spacing did not lead to an increase in peripheral reading speed, though exaggerated word spacing did. The former may not have succeeded because any advantage gained by minimizing the effects of letter crowding were negated by a reduction in word shape information, a factor known to be critical for efficient reading^40^. The benefits of exaggerated vertical word spacing, on the other hand, were assumed to reflect a reduction in crowding. This conclusion is supported by Blackmore-Wright *et al*.^37^, who examined the possible interactive effects of vertical and horizontal word spacing on reading performance in both visual normals and individuals with macular degeneration. Nonetheless, even with exaggerated word and line spacing, improvements in reading speed tend to be modest for individuals with moderate to severe central visual loss (see Fig. 7 in Blackmore-Wright *et al*.^37^).

Our principal aim in this paper was to develop a peripheral reading protocol that eliminates the effects of spatial crowding altogether, but does so without introducing the confounding effects of temporal crowding. This was done by presenting each word in isolation but in a position that mimics its natural position in the line of text being read. Our protocol, which we term *Word Mode*, allows for a near-normal pattern of eye movements and takes into consideration individual differences in the temporal processing speed of peripherally-viewed text. We quantified the effects by Word Mode by: (i) measuring peripheral reading speed and word accuracy for a range of text sizes; and (ii) comparing how the pattern of reading eye movements differ between Word Mode and normal text.

## Results

### Reading speed

**Figure 1:**
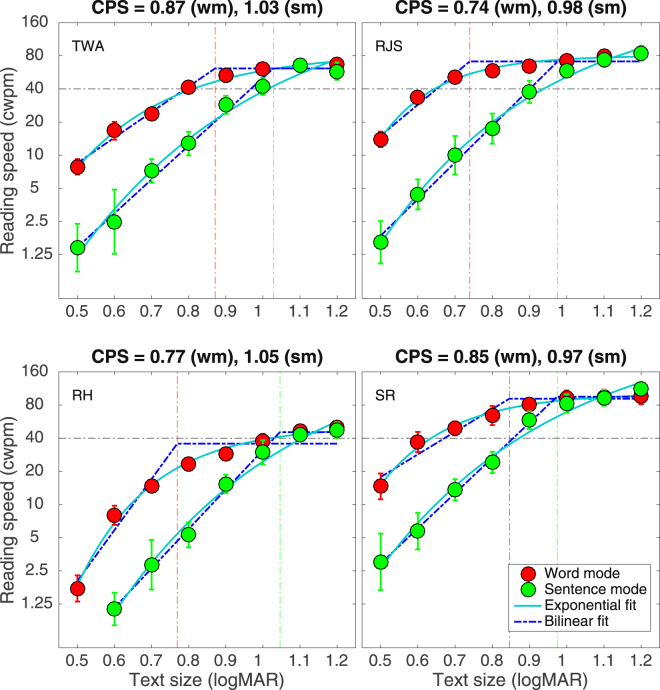
Mean reading speed (correct words per minute ± 84% CI) as a function of text size (logMAR) for four observers, for both Word Mode (red circles) and Sentence Mode (green circles). Blue dashed line through each data set show the best fit of a bilinear function (see Supplementary Material for details). The elbow of the bilinear functions, demarcated by vertical red (Word Mode, wm) and green (Sentence Mode, sm) dashed vertical lines, specifies the critical print size (CPS), which is reported at the top of each panel. Solid blue line through each data set show the best fit of an exponential function (see Supplementary Material for details). Note that observer RH was unable to read any words in Sentence Mode at a text size of 0.5 logMAR.

Reading speeds, defined as the number of correctly read words per minute, were averaged across sessions for each observer. Figure 1 shows the mean reading speeds as a function of text size for each observer (separate panels) and each presentation mode. The vertical error bars show the 84% confidence intervals (CIs), which allow a direct visual comparison of responses across experimental conditions with an alpha of approximately 0.05 (i.e. non-overlapping error bars denote a significance difference at the 0.05 level)^41,42^. The Word Mode protocol resulted in significantly faster reading speeds than Sentence Mode for all text sizes up to approximately 1.0 logMAR, above which reading speeds were approximately the same for both modes. At the smallest text size that could be read in both experimental modes (0.6 logMAR), when averaged across observers, Word Mode yielded faster reading speeds than Sentence Mode by a factor of 7.1.

The solid and broken blue functions through each data set show the least-squares fit of an exponential function and bilinear function, respectively (see Supplementary Information for details of fitting procedure, statistical analysis of each fit, and justification for functions chosen). Following Legge *et al*.^43^, the bilinear functions were used to determine critical print size, defined as the smallest print size below which reading speed begins to decline sharply^44^. Averaged across all observers, critical print size for Word Mode was less than that for Sentence Mode by 0.20 logMAR.

The horizontal broken line in each panel shows the minimum acceptable speed (40 wpm) for reading short information messages, termed ‘spot reading’^45,46^. A spot reading speed was achieved for a text size of 0.78 logMAR with Word Mode and 0.98 logMAR with Sentence Mode (averaged across observers, and determined from the fit of the exponential functions). Note that this acuity difference for spot reading (0.20 logMAR) matches the critical print size difference between Word Mode and Sentence Mode that was derived from the fitted bilinear functions.

**Figure 2:**
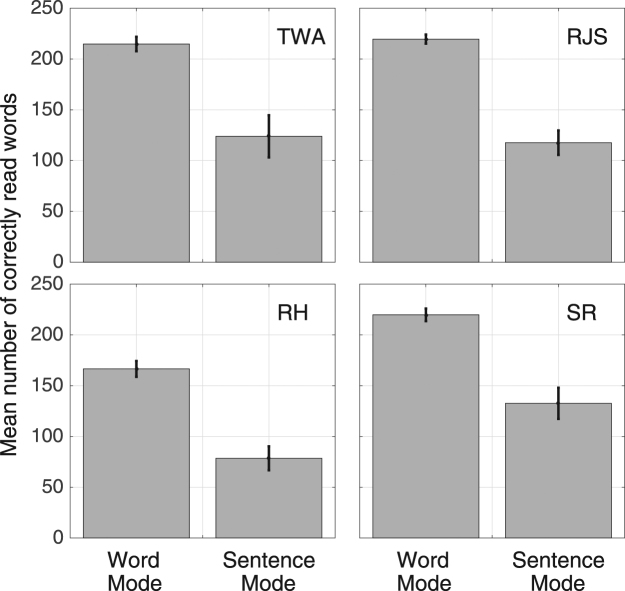
Reading accuracy for Word Mode and Sentence Mode, showing the mean number of correctly read words for all text sizes less than 1.0 logMAR, averaged across four observers. An accuracy of 100% is equivalent to 250 correctly read words. The vertical error bars show ± 84% confidence intervals.

Figure 2 shows, for both reading modes and for four observers, the mean number of correctly read words for all text sizes less than 1.0 logMAR, where the reading speed between Word Mode and Sentence Mode differ significantly (see Fig. 1). Note that, for all observers, reading was significantly more accurate in Word Mode than Sentence Mode.

**Figure 3:**
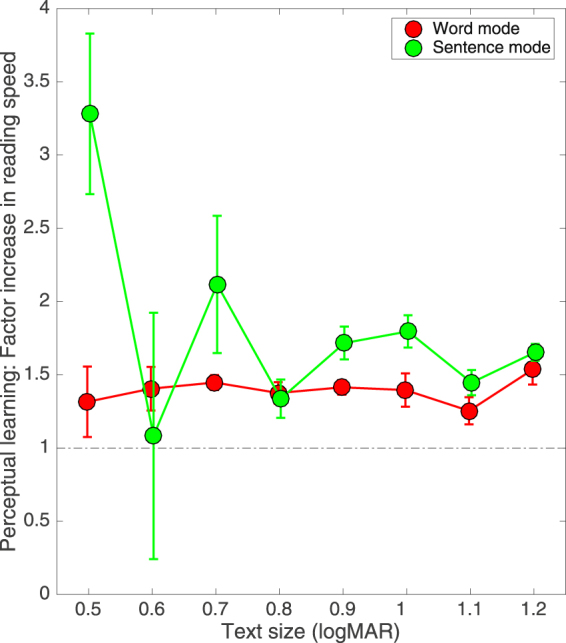
Increase in reading speed, calculated as the ratio of the first two experimental sessions (averaged) divided by the last two sessions (averaged). Results, averaged across four observers, are shown for both Word Mode (red circles) and Sentence Mode (green circles) as a function of text size (logMAR). Error bars (±84% CI) represent inter-observer variability.

To allow for practice effects and perceptual learning, observers were allowed two practice sessions prior to the collection of experimental data (see Methods). Nonetheless, we observed that a degree of perceptual learning was evident in each observer over the course of eight experimental sessions. Figure 3 shows the ratio of reading speeds, calculated as the mean speed for the last two sessions divided by the mean speed for the first two sessions, averaged across observers. Results are plotted as a function of text size for each text presentation mode. As defined here, the extent of perceptual learning for Sentence Mode was greater than or equal to that for Word Mode at all text sizes employed. This provides evidence that the extent of perceptual learning was closer to plateau performance in Word Mode than Sentence Mode.

### Eye movements

**Figure 4:**
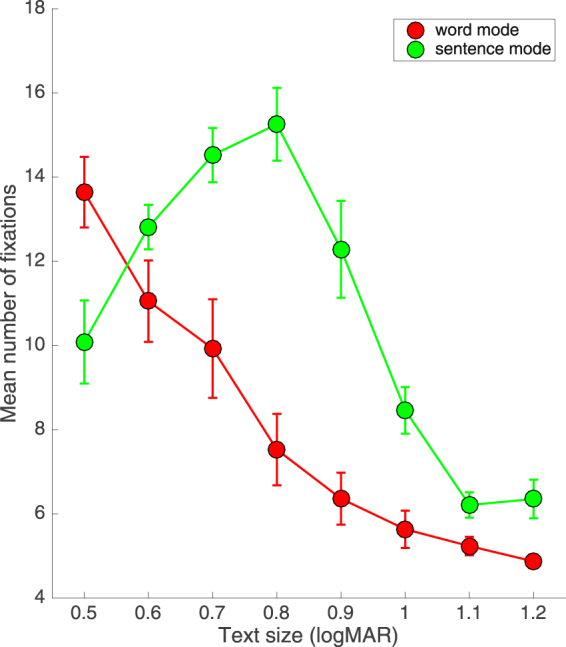
Average number of fixations per sentence, averaged across all four observers, as a function of text size (logMAR) for both Word Mode (red circles) and Sentence Mode (green circles). Error bars (±84% CI) represent inter-observer variability.

The gaze position data revealed different eye fixation patterns between Word Mode and Sentence Mode. Figure 4 shows the mean number of fixations per sentence, averaged across observers, as a function of text size. The vertical error bars show ±84% confidence limits. In Word Mode the number of fixations declined smoothly as text size increased from 0.5 to 1.2 logMAR, whereas in Sentence Mode the number of fixations was maximal for intermediate text sizes (~0.8 logMAR). Note that, with the exception of the smallest text size employed (0.5 logMAR), the mean number of fixations per sentence read was significantly greater for Sentence Mode than Word Mode.

**Figure 5:**
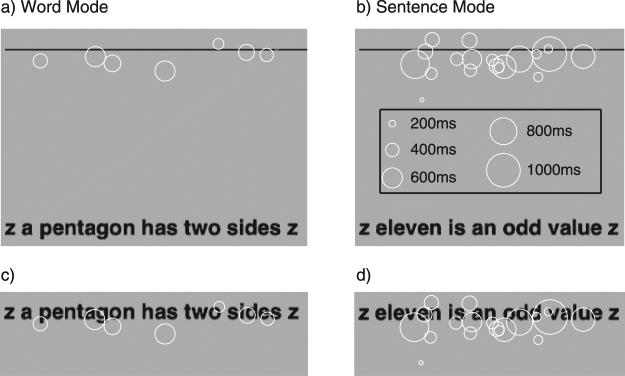
Fixation patterns for observer RJS, for both Word Mode (panels a, c) and Sentence Mode (panels b, d), reading 0.8 logMAR text. The top panels show the fixation line (solid horizontal line), the number of fixations (as white circles of varying diameter to indicate fixation length in ms) and the target sentence. The separation between the fixation line and the target sentence is representative of the viewing display used. Note that, for comparison, the whole sentence is shown in Word Mode. In panels (c) and (d) the text has been raised vertically onto the centre of the fixation line, and the fixation line removed.

The largest deviation in the number of fixations between Word- and Sentence-mode occurred for a text size of 0.8 logMAR. Figure 5 shows, for this text size, typical fixation patterns for one observer (RJS). In panels (5a) and (5b) the separation between the fixation line and the target sentence is representative of the viewing display used. To allow comparison with foveal reading, in panels (5c) and (5d) the text has been raised vertically to where the fixation line was situated. Note that multiple fixations were required for peripheral reading in Sentence Mode, with several scattered about each word. However, in Word Mode there were fewer fixations, yielding a more coherent fixation pattern that is broadly similar to that observed with foveal reading.

**Figure 6:**
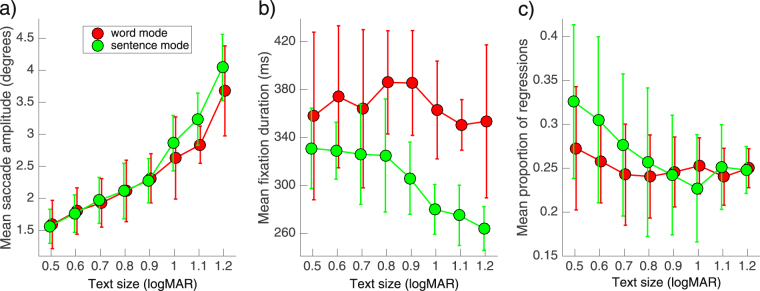
Saccadic amplitude (panel a), fixation duration (panel b) and proportion of eye movement regressions (panel c) plotted as a function of text size from 0.5 to 1.2 logMAR. The symbols show the mean across four observers for both Word Mode (red circles) and Sentence Mode (green circles). The vertical error bars (±84% CI) represent inter-observer variability.

From the eye movement recordings, we also examined saccade amplitude, fixation duration and the proportion of regressions for both Word Mode and Sentence Mode. The mean results across four observers are shown in Fig. 6. Note that there were no significant differences in either mean saccadic amplitude or the proportion of regressions between the two text presentation modes. However, for text sizes above 0.8 logMAR the mean fixation duration was less for Sentence Mode than Word Mode by approximately 80 ms.

## Discussion

Our aim in this paper was to develop a reading protocol for peripheral vision that is free from the disruptive effects of both spatial^32,34,37^ and temporal crowding^24,25^, and tolerates individual differences in the temporal processing speed of eccentrically-viewed text^26^. This was achieved by presenting each word in isolation but in a position that mimicked its natural position in the line of text being read, with each new word elicited using a self-paced button press. To help our naïve observers read with peripheral vision, a fixation line was present in all conditions and we acknowledge this may have had a stabilizing influence on eye movements. Nonetheless, compared with reading whole sentences, our crowding-free protocol – termed Word Mode – increased reading speeds in peripheral vision by up to a factor of seven (Fig. 1), resulted in significantly fewer reading errors (Fig. 2) and fixations per sentence (Fig. 4), and reduced both the critical print size and the text size required for spot reading by 0.2–0.3 logMAR (Fig. 1). We suggest that the level of reading efficiency afforded by Word Mode may return reading as a viable activity to many individuals with macular disease.

Although Word Mode was superior to Sentence Mode in terms of reading accuracy and mean number of fixations, both modes yielded similar estimates of reading speed at text sizes above 1.0 logMAR. However, it is important to consider that our ‘normal text’ (Sentence Mode) measures were completed using single line, five-word sentences. Therefore, our Sentence Mode measures were not subject to the known detrimental effects of line crowding^34,37^. Blackmore-Wright *et al*.^37^ reported that, with eccentric viewing, line crowding was in fact greater than the word crowding when reading a full paragraph of large print text (>1.2 cps). Given this, we anticipate that reading speed for text sizes above 1.0 logMAR would be greater for Word Mode than for multi-lined paragraphs because the former necessarily eliminates both word *and* line crowding.

Readers with central vision impairment have a greater number of fixations and shorter fixation durations than normally sighted readers^47^, with fixations clustered around words that are difficult to read^48^. Such fixation clusters may, at least in part, be attributed to a word frequency effect^48^, where infrequent words trigger more fixations. An alternative explanation for fixation clusters is that the reader may be attempting to ‘uncrowd’ a word whose letters are relatively crowded because of longer word length and/or because the word contains more closely spaced letters (as may occur in a proportional font). It is known that word recognition, and hence reading speed, is affected by letter crowding^32,33^.

The eye movement data shown in Fig. 4 are consistent with a crowding-based account of peripheral reading, in which the number of fixations varies depending on the degree of word and/or letter crowding. Reading in Word Mode required approximately one fixation per word for text sizes ≥1.0 logMAR; below 1.0 logMAR the number of fixations steadily increased as text size decreased. As word crowding was absent for this reading protocol, the increasing number of fixations with decreasing text size may reflect letter crowding and/or attempts by the reader to uncrowd the letters through image jitter^49^.

Reading in Sentence Mode was subject to the effects of both letter crowding and word crowding. We assume the different number of eye fixations between Word and Sentence Modes depicted in Fig. 4 reflects the effects of word crowding. Note that, except for the smallest text size employed (0.5 logMAR), the number of fixations required for reading five words was significantly greater in Sentence Mode than Word Mode. Sentence Mode yielded fewer fixations at the smallest text size presumably because more than one word could be read in a single fixation, something that was not possible in Word Mode.

From our eye movement data, we also assessed saccade amplitude, fixation duration and the proportion of regressions for each text presentation mode, as such measures have proven beneficial in examining more cognitive aspects of reading^50^. While we observed no significant differences between modes for either saccadic amplitude or the proportion of regressions, we did observe a significant reduction in fixation duration for Sentence Mode in comparison with Word Mode for text sizes above 0.8 logMAR (see Fig. 6). Taken overall, our eye movement data support the view that the faster reading speed afforded by Word Mode largely reflects the reduced number of fixations required to read a full sentence in that mode (Fig. 4).

Based on the results reported here, we suggest that future word crowding investigations would best be undertaken at text sizes below the critical print size, where word crowding appears to be greatest (Fig. 1). This conclusion is supported by Chung^34^, who found the largest effects of vertical word spacing for text sizes less than the critical print size. Similarly, in a letter crowding investigation, Haberthy & Yu^51^ found that sequentially presenting a word’s component letters increased peripheral reading speeds for text sizes below but not above the critical print size. The use of text that was solely above the critical print size may have diminished the possibility of discovering differences between experimental conditions in the word-crowding study by Yu *et al*.^36^.

Finally, Word Mode was primarily designed for reading with peripheral vision, where crowding can occur across the whole of the text being read. Thus, Word Mode will be of primary benefit to readers with central vision loss. However, it may also benefit readers with certain types of dyslexia^52–56^ or amblyopia^57–60^, because they experience crowding in central vision. We believe our Word Mode protocol is ideally suited to an app for SmartScreen displays, whereby a simple screen tap triggers the presentation of each successive word. Such an app would be useful for both digital reading material and a photograph of printed material.

## Methods

### Participants

There were four participants, aged 16 to 39 years. One (RJS) was a highly-experienced psychophysical observer, though with no previous experience of reading with non-central vision. The other participants had no visual psychophysical experience. All participants had normal visual fields and corrected-to-normal acuity, and all gave informed consent. In addition, parental consent was obtained for the one participant aged less than 18 years. Participant RH did not have English as a first language, but had been living and working in the UK for many years and was a fluent reader and speaker of English. The other three were native English speakers. The study was approved by the Aston ethics committee and adhered to the tenets of the declaration of Helsinki.

### Stimuli

Short, true or false mathematical statements were displayed at a viewing distance of 55 cm on a Nokia monitor at a frame rate of 100 Hz using a HP Compaq pc and the Eyelink Matlab toolbox^61^. Eight statement categories were used and they are listed in Table 1, together with examples of true and false statements for each category. The text was rendered in Helvetica font and was presented at eight text sizes (defined as the height of a lower case x) in a logarithmic progression from 0.5 to 1.2 logMAR (0.26 to 1.32 degrees). The text was presented at maximum contrast as black (contrast 0.9998, 0.01 cd/m^2^) on a mid-grey background (45.00 cd/m^2^). The Weber definition of contrast was applied.

**Table 1:** Examples of the true and false mathematical statements used to assess reading performance. Each statement consisted of five words.

Statement category	True statement	False statement
Multiplication	one times nine is nine	two times three equals four
Addition	four plus four equals eight	four plus three is five
Subtraction	fifteen minus six equals nine	six minus three equals two
Division	four into four goes once	ten into one goes twice
Comparison	eight is more than six	nine is less than six
Square/root-determination	ten squared is one hundred	root of four equals one
Even/odd-determination	three is an odd number	eight is an odd integer
Polygon-determination	a rectangle has four sides	a pentagon has three angles

The statements were presented at a retinal eccentricity of 7.5^°^ in the inferior visual field, and each was read by saccading along a fixation line. Eccentricity was measured as the vertical distance from the fixation line, which was present throughout all trials, to the centre of the statement (defined as the centre of the x-height). The grey fixation line (contrast 0.25) was two pixels wide, centred about the vertical midline of the screen and of the same length as the longest statement at the presented text size. Each statement was presented entirely in lower case and was flanked at its ends by a lower case z in order to ‘crowd’ the first and last words. The statements (with flankers) were aligned with the left end of the fixation line.

In each session, the statements were presented either as a full sentence (termed Sentence Mode) or one word at a time (termed Word Mode). In Sentence Mode, the observer pressed a mouse button to make each sentence appear at the centre of the screen. In Word Mode, the button press triggered the display of each word, but the words remained in their Sentence Mode position. We did not assess the effect of multiple button presses *per se* on reading speed and therefore, logically, we cannot distinguish between the benefit afforded by uncrowded text and the effect of multiple button presses. We assumed that the requirement to press the mouse button five times per sentence (Word Mode) would be unlikely to affect an increase in reading speed compared with one button-press per sentence (Sentence Mode).

**Figure 7:**
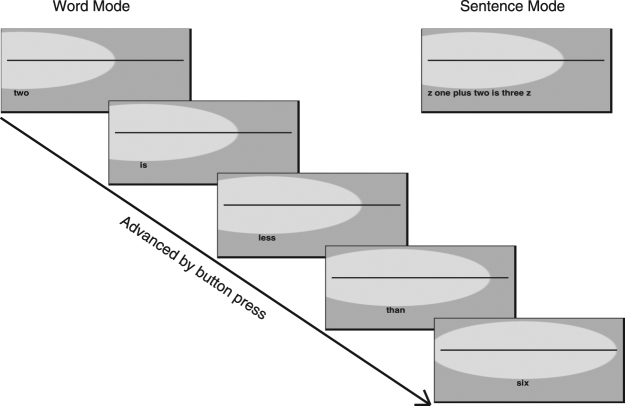
Schematic of experimental protocol (approximately to scale for the largest text size used, but with the lower part of the screenshots truncated), for one trial of both Word Mode and Sentence Mode. On the experimental display monitor, the simulated scotoma (shown here as a light grey ellipse) was the same luminance and colour as the background (shown here as mid-grey). In this schematic, a true statement is shown for both modes.

A gaze-contingent scotoma paradigm was employed throughout the experiment: an Eyelink 1000 eye tracker was used to monitor eye position and to generate an artificial scotoma centered on fixation, covering the text (but not the fixation line) with mid grey. The height of the artificial scotoma was twice the distance between the upper boundary of the text and the fixation line, and its width was three times its height (see Fig. 7). It had a central plateau of zero transparency spanning 95% of its width and height, which smoothly transitioned to 100% transparency at its border with a half cycle cosine profile. A saccade to any visible letter caused the entire sentence to be obscured by the artificial scotoma for all except the largest text size, for which the start or end of the sentence may have been partly visible. The observer’s dominant eye was used for tracking. To prevent the observer from seeing part or all of the text by fixating well below the fixation line and making a blink^62^, we constrained the vertical position of the artificial scotoma’s centre to be no lower than the centre of the text.

### Procedure

Reading speeds were determined for each text size and presentation mode. Every trial was initiated by a button press and consisted of a single-interval presentation of the target statement or word, which was read aloud by the observer. Subsequent button presses immediately replaced the current text (sentence or word) with the next text. Thus, in Sentence Mode only one button press was required per statement, whereas in Word Mode five presses were required per statement. Figure 7 shows a schematic of the experimental protocol. In every session, each sentence was selected at random, without replacement, from a bank of 300 different statements (150 true and 150 false statements). Viewing was binocular, with the observer’s head stabilized using a chin and forehead rest, with additional restraints to the top and sides of the head. Responses were audio-recorded and scored off-line to assess reading accuracy (i.e. to determine the number of correctly read words). For both Sentence Mode and Word Mode, reading speed was calculated by dividing the number of words that were read correctly by the time taken to read each full sentence (derived from the button-press time stamps).

In each session the sentences were displayed at all eight text sizes and in both modes, thus forming 16 blocks. Each block consisted of 10 sentences at a given text size and presentation mode, which the observer read aloud as quickly and accurately as possible. Every block began with an eyetracker calibration and validation routine, using nine onscreen targets. Calibration and/or validation were repeated until the validation error was less than 1° on average, with a maximum error allowed for any one point of 1.5°.

The end of each block was announced by an audio signal. Observers were free to rest at the end of each block as required. In each session, the text size order was randomized using a Latin square, which ensured that the text size order was counterbalanced across all eight sessions. The presentation mode alternated between blocks and its identity was announced at the start of each block. The presentation mode that appeared first varied across sessions in a pseudo-random order. The eight data-collection sessions were preceded by two practice sessions, whose data are not included in analysis.

### Data Availability

The datasets generated during the current study are available in the Aston Data Explorer repository [http://doi.org/10.17036/researchdata.aston.ac.uk.00000282].

## Electronic supplementary material

Supplementary information
