# Supplementary material for: Word Mode: a crowding-free reading protocol for individuals with macular disease
Source: Sci Rep. 2018 Jan 19;8:1241. doi: 10.1038/s41598-018-19859-0 (PMC5775436; doi:10.1038/s41598-018-19859-0)
Supplement: Supplementary file 1 — Supplementary information [file 41598_2018_19859_MOESM1_ESM.pdf]

Supplementary Information file

Title: Word mode: a crowding-free reading protocol for individuals with macular disease

Authors: Stuart Wallis[1*], Yit Yang[2] & Stephen J Anderson[1]

[1]School of Life & Health Sciences, Aston University, Birmingham B4 7ET, UK
[2]Royal Wolverhampton NHS Trust, New Cross Hospital, Wolverhampton WV10 0QP, UK

Supplementary Information

The blue dashed lines in Fig. 1 are bilinear functions, given by:

$$\log(y) = \min(mx + C_1, C_2),$$

where m is the left line's gradient, C_1 is its intercept, and C_2 is the right line's vertical position (it has zero gradient). The functions were fitted to the data using *fminsearch* in Matlab to minimize the sum squared error between function and data. A bilinear function was chosen because previous research has demonstrated that reading speed versus text size data is readily characterized by a linear increase in reading speed with increasing text size until a plateau is reached at the critical print size (cps) – above this text size, no further increase in reading speed is observed[1,2,3]. Using normal text (i.e. Sentence Mode), our observers had a mean cps of 1.01 logMAR, which falls within the range of cps values reported at similar eccentricities for normally-sighted observers[2,4].

However, the disparity between the fitted function and the red symbols in Fig. 1 show that the word mode reading speeds for RJS and SR are not well-described by a bilinear function. An alternative is to use an exponential function[5]. The solid lines in Fig. 1 represent the best fit (least squares) of an exponential decay function given by:

$$\log(y) = m * (1 - e^{-c(x - s)})$$

where m is the asymptotic reading speed, c is the rate of reading speed change with print size, and s is the print size at which reading speed is 0 log (cwpm).

In Sentence Mode, the total sum squared error was smaller for the bilinear function (0.068) than the exponential function (0.220), but in Word Mode the reverse was true (bilinear 0.169; exponential 0.027). F-ratio tests were conducted separately for each observer and mode (see Table S1). In Word Mode, the exponential function provided a better fit than the bilinear function for all observers, and this reached statistical significance at the 0.05 level for three observers. In Sentence Mode, the bilinear function provided a better fit than the exponential function for all observers, but this reached statistical significance only for observer RJS (see Table S1).

	Word Mode			Sentence Mode		
Observer	F	p (exponential fits better)	df	F	p (bilinear fits better)	df
TWA	0.556	0.247	6	1.919	0.224	6
RJS	0.158	0.021	6	4.513	0.045	6
RH	0.111	0.009	6	4.123	0.054	5
SR	0.164	0.022	6	2.673	0.128	6

Table S1*. F-ratio test results for each observer and text presentation mode.*

Supplementary references

1. Mansfield, J. S., Legge, G. E. & Bane, M. C. Psychophysics of reading. XV: Font effects in normal and low vision. *Invest. Ophthalmol. Vis. Sci.* **37,** 1492–1501 (1996).

2. Chung, S. T. L. Reading speed benefits from increased vertical word spacing in normal peripheral vision. *Optom. Vis. Sci.* **81,** 525–535 (2004).

3. Martelli, M., Filippo, G. D., Spinelli, D. & Zoccolotti, P. Crowding, reading and developmental dyslexia. *J. Vis.* **9,** 1-18 (2009).

4. Chung, S. T. L. The effect of letter spacing on reading speed in central and peripheral vision. *Invest. Ophthalmol. Vis. Sci.* **43,** 1270–1276 (2002).

5. Cheong, A. M. Y., Legge, G. E., Lawrence, M. G., Cheung, S. & Ruff, M. A. Relationship between slow visual processing and reading speed in people with macular degeneration. *Vision Res.* **47,** 2943–2955 (2007).
